# Retraction Note: Auricular cartilage regeneration using different types of mesenchymal stem cells in rabbits

**DOI:** 10.1186/s40659-026-00737-3

**Published:** 2026-10-20

**Authors:** Taghreed Ahmed Hassan, Mohamed Ahmed Maher, Amr Fekry El Karmoty, Zainab Sabry Othman Ahmed, Marwa A Ibrahim, Hamdy Rizk, Ayman Tolba Reyad

**Affiliations:** 1 Anatomy and Embryology Department, Faculty of Veterinary Medicine, Cairo University, Giza Square, Giza, 12211 Egypt; 2 Cytology and Histology Department, Faculty of Veterinary Medicine, Cairo University, Giza, Egypt; 3 King Salman International University, South Sinai, Ras Sudr, Egypt; 4 Department of Biochemistry and Molecular Biology, Faculty of Veterinary Medicine, Cairo University, Giza Square, Giza, 12211 Egypt

**Retraction Note to: Hassan et al. Biological Research (2022) 55:40** **https://doi.org/10.1186/s40659-022-00408-z**

The Editors-in-Chief have retracted this article. After publication, several concerns were raised regarding the figures. In figure 3 ADMSCs panel A appears to overlap with EMSCs panel A with frameshift, and ADMSCs panel B appears to overlap with EMSCs panel B with frameshift. BMSCs panel B appears to overlap with rotation with another article published later by some of the same authors [1]. In figure 4, the same control images were used for all four experimental conditions and therefore do not correspond to the images in each time course. The authors were unable to provide sufficient raw data or a sufficient response to concerns. The alternative images provided were not conducted at the time of the experiment, or could not be validated for correction. The Editors have lost confidence in the data and conclusions of this article.

Taghreed Ahmed has stated on behalf of all authors that the authors disagree with this retraction.
